# A Case of Untreated Myeloid Sarcoma of the Pancreas Head Region: Diagnostic Process of AML Subtyping in an Autoptic Case

**DOI:** 10.1155/2021/7439148

**Published:** 2021-12-21

**Authors:** Yuki Fukumura, Gentaro Taniguchi, Ai Koyanagi, Yuki Horiuchi, Tomonori Ochiai, Yoko Tabe, Katsuhiro Sano, Yifare Maimaitiaili, Naomi Otsuji, Karin Ashizawa, Takashi Yao

**Affiliations:** ^1^Department of Human Pathology, Juntendo University, School of Medicine, Tokyo, Japan; ^2^Department of Gastroenterology, Juntendo University, School of Medicine, Tokyo, Japan; ^3^Department of Pathology and Oncology, Juntendo University, School of Medicine, Tokyo, Japan; ^4^Department of Clinical Laboratory Medicine, Juntendo University, School of Medicine, Tokyo, Japan; ^5^Department of Hematology, Juntendo University, School of Medicine, Tokyo, Japan; ^6^Department of Radiology, Juntendo University, School of Medicine, Tokyo, Japan

## Abstract

This study describes an autopsy case of pancreatic/peripancreatic myeloid sarcoma in a 70-year-old man, initially presenting with obstructive jaundice. Pathologically, diffuse infiltration of round cells containing atypical nuclei with marked cleavage was observed mainly in the pancreas head, peripancreatic lymph nodes, spleen, bilateral lung, and bone marrow. Immunohistochemically, the tumor cells were negative for CD20, CD79a, CD3, CD5, c-kit, CD34, and TdT and positive for myeloperoxidase, CD33, CD68, and CD163. Flow cytometry of the peripheral blood showed underexpression of CD11c and aberrant expression of CD56 in the monocyte subset. The peripheral blood smear showed an increase in monocytes and atypia in neutrophils and monocytes, as well as enlarged platelets and polychromatic erythroblasts. Hence, it was suggested that the myeloid sarcoma was derived from the acute transformation of chronic myelomonocytic leukemia. Myeloid sarcoma is an extramedullary-mass-forming hematologic malignancy that is difficult to diagnose, especially when the initial presentation is a pancreatic mass. However, early diagnosis is important for appropriate therapy. Although bone marrow examination could not be performed because of the patients' severe condition, the pathological specimen obtained with autopsy helped subtype the patient's leukemia. The immunohistochemical features of this case merit attention.

## 1. Introduction

Myeloid sarcoma (MS) is a rare disease characterized by a tumor mass consisting of immature myeloid cells at an extramedullary site [1]. MS may develop de novo or may be associated with acute myeloid leukemia (AML) or other hematologic malignancies [2]. Because of its rarity, MS is difficult to diagnose, especially when an MS mass is the first presentation in the absence of a previous hematologic disorder. Although almost any site of the body can be affected by MS, the most frequently involved sites are the skin and lymph nodes, while pancreatic involvement is rare. To our knowledge, only around five MS cases in which the first presentation was a pancreatic mass have been reported in the English literature [3].

It was highly suspected that the patient had chronic myelomonocytic leukemia (CMML) in the background of MS because of the peripheral blood findings. CMML is a rare clonal hematopoietic malignancy with features of myeloproliferative neoplasm (MPN) and myelodysplastic syndrome (MDS) [4, 5].

Herein, we present an autopsy case of MS of the pancreas head region possibly derived from acute transformation of CMML in a patient whose initial presentation was obstructive jaundice due to pancreatic mass. He had never previously been diagnosed with hematologic malignancy. He died in a week after his administration to our hospital with disseminated intravascular coagulation (DIC) due to a pulmonary tumor thrombotic microangiopathy- (PTTM-) like condition.

## 2. Case Presentation

A 70-year-old man who was previously healthy visited our hospital because of yellowing of the skin and conjunctiva that started 7 days before his visit to our hospital. He had not undergone a health checkup in the last 15 years and was previously a heavy drinker but not a smoker.

### 2.1. Days 1-4 from Admission

**Figure 1:**
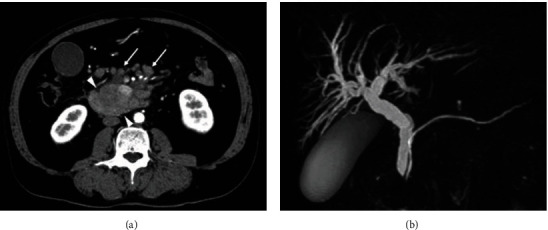
(a) Computed tomography image (pancreatic parenchymal phase). A slightly enhanced, relatively homogeneous tumor can be seen in the pancreas head (arrowheads). Multiple enlarged peripancreatic and mesothelial lymph nodes (arrows) are visible. (b) Magnetic resonance cholangiopancreatography image showing bile duct dilatation.

Laboratory tests at the time of admission showed the following results: hemoglobin, 13.6 g/dL; white blood cell (WBC) count, 12,000/mm^3^, platelet (PLT) count, 119 × 10^3^/mm^3^; aspartate aminotransferase, 135 U/L; alanine aminotransferase, 211 U/L; lactate dehydrogenase, 762 U/L; total bilirubin, 9.01 mg/dL; direct bilirubin, 6.79 mg/dL; and C-reactive protein (CRP), 1.76 mg/dL. Abdominal computed tomography (CT) with enhancement showed a 44 mm, gradually enhanced mass with multiple enlarged lymph nodes, including peripancreatic and mesenteric nodes (Figure 1(a)). Magnetic resonance cholangiopancreatography (MRCP) revealed dilatation of the common bile duct (Figure 1(b)). CT did not show splenomegaly or hepatomegaly, and no metastatic lesions were identified in the liver or lungs. Serological tests for tumor markers showed the following results: carcinoembryonic antigen, 3.2 ng/mL; carbohydrate antigen, 19-9, 67 U/mL; and Span-1, 37 U/mL.

For bilirubin reduction, endoscopic retrograde cholangiopancreatography was initially performed, but due to compression by the tumor, the ampulla of Vater was not detected; hence, a hepatogastrostomy was performed. Tumor biopsy was performed from the periampullary site; however, no tumor cells were observed on pathological evaluation.

### 2.2. Days 5-8 from Admission

**Figure 2:**
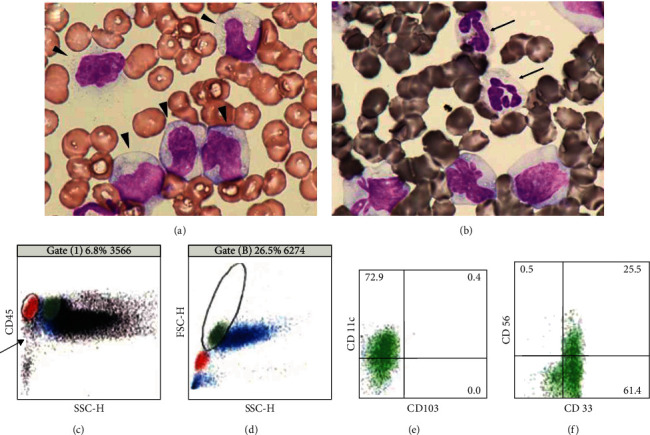
Increased (a, arrowheads) monocytes and neutrophils with degranulation/hypogranulation in which (b, arrows) MPO shows negative or weakly positive in peripheral blood ((a) May-Grünwald stain, (b) myeloperoxidase stain). Flow cytometry of the peripheral blood shows a few scattered blastoid cells with CD45/side scatter gating (c, arrow). (d) With forward versus slide scatter gating, (e) underexpression of CD11c and (f) aberrant expression of CD56 are shown for monocyte subset.

Although the patient was in relatively good condition until the 4th day from admission, he developed fever of 38.1°C on the 5th day. His laboratory test showed a WBC count of 27,000/mm^3^ and CRP level of 2.1 mg/dL, and he developed respiratory failure. An increase in monocytes (>1 × 10^9^/L), atypical neutrophils, enlarged PLTs, polychromatic erythroblasts, and atypical monocytes were seen in the peripheral blood (Figures 2(a) and 2(b)); hence, hematological malignancy was suspected and a bone marrow examination was planned. However, his severe respiratory condition did not permit this procedure. Flow cytometry of the peripheral blood was performed, showing a few blastoid cells with CD45/side scatter gating and aberrant expression of CD56/underexpression of CD11c for monocyte subsets with forward versus slide scatter gating (Figures 2(c)–2(f)). He died of respiratory failure on the 8th day of admission, 14 days from the initial presentation of skin yellowing and conjunctiva. Subsequently, an autopsy was performed.

### 2.3. Pathological Findings

**Figure 3:**
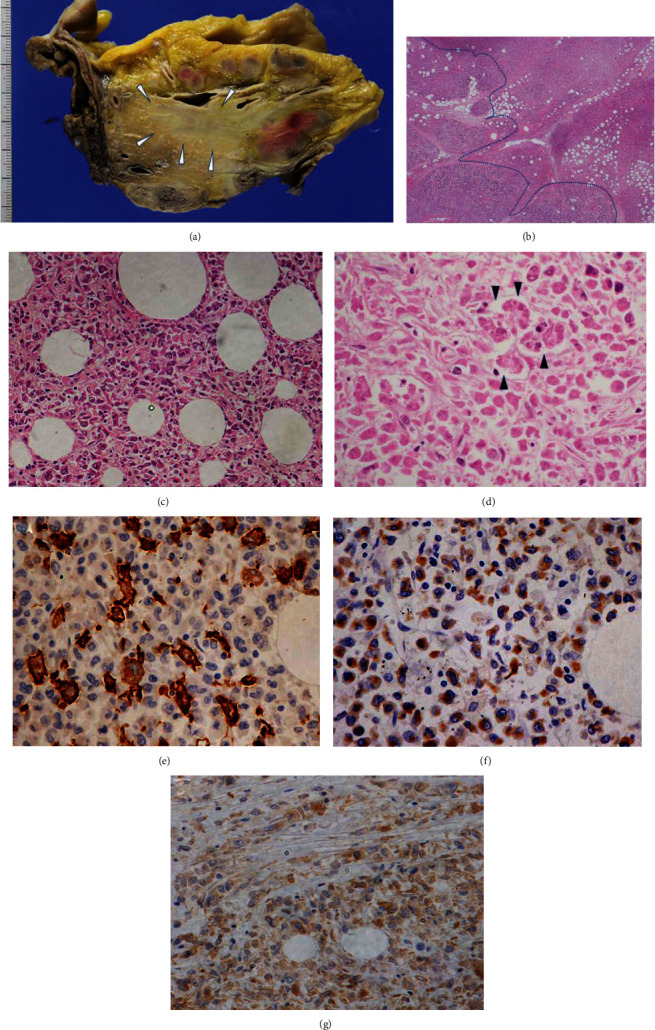
(a) Cut section of the pancreatic head showing an ill-circumscribed whitish mass (arrowheads) and multiple swollen peripancreatic lymph nodes. Microscopic image showing a (b) rather circumscribed tumor border (dotted line) and (c) discohesive and round tumor cells, often with marked cleaved nuclei. Tumor cells with remaining scattered pancreatic acinar cells (arrowheads) are seen (d). Immunohistochemistry showing some tumor cells are positive for (e) CD163 and most tumor cells are positive for (f) myeloperoxidase and (g) CD33 (immunohistochemistry for (e) CD163, (f) myeloperoxidase, and (g) CD33).

During the autopsy, a whitish mass was found, involving the pancreatic head and peripancreatic lymph nodes (Figure 3(a)). Microscopic examination revealed diffuse infiltration of the mass by round cells containing atypical nuclei with marked cleavage (Figures 3(b)–3(d)). On immunohistochemical analysis, the tumor cells were completely negative for AE-1/-3, CAM5.2, CD20, CD79a, CD3, CD5, c-kit, CD34, and TdT. Most tumor cells were positive for myeloperoxidase and CD33 expression. Some, but not all, tumor cells were positive for CD68 and CD163 (Figures 3(e)–3(g)). Based on these findings, we diagnosed the pancreatic mass as MS composed of myelocytic lineage blasts. Along with the findings in the patient's peripheral blood and flow cytometry data, this MS was suggested to be derived from acute transformation of CMML.

**Figure 4:**
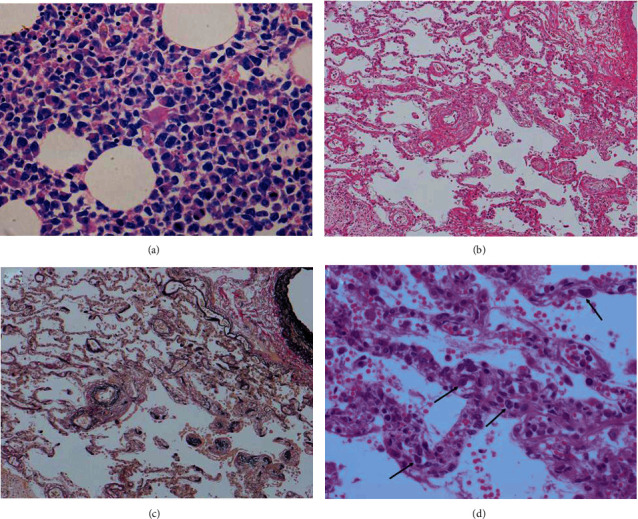
(a) Markedly hypercellular bone marrow with discohesive, round tumor cells similar to those seen in the pancreatic head. Tumor cell emboli at alveolar wall with fibrous thickening of pulmonary vasculature (b, c). Leukemic cells are indicated by the arrows (d).

In addition to the pancreas head, a few small MS masses with similar cell composition were detected in the intestinal wall, in fat tissue posterior to the left kidney, and in the thyroid. The bone marrow showed a marked hypercellular state in which >50% of the cells were the same as those seen in the pancreatic head (Figure 4(a)). The tumor cells also diffusely infiltrated the spleen and were scattered throughout the liver sinusoid. In both lungs, tumor emboli were observed in the capillaries, small vessels, and small arteries. Diffuse fibrotic thickening of small veins and interalveolar fibrinous exudates were observed, suggesting a PTTM-like condition (Figures 4(b)–4(d)).

## 3. Discussion

MS, also known as granulocytic sarcoma or chloroma, occurs in association with diverse hematologic diseases. Classically, MS is mostly associated with the AML subtypes of French-American-British (FAB) M1 (AML without maturation) and M2 (AML with maturation), whereas recent reports have shown that the M4 (acute myelomonocytic leukemia) and M5 (acute monoblastic and monocytic leukemia) subtypes are also frequent [3, 6]. The detection of MS is considered equivalent to a diagnosis of AML.

We considered the patient had CMML in the background of MS, based on the following three findings: [1] monocytosis and multilineage dysplasia seen in the peripheral blood, [2] aberrant expression of CD56 and underexpression of CD11c in the monocyte subset seen in the flow cytometry of the peripheral blood, and [3] the microscopic and immunohistochemical features of the tumor cells observed in autopsy. The aberrant expression of CD56/underexpression of CD11c in the monocyte subset has been reported to be fairly/highly specific for CMML, respectively [7, 8]. The microscopic and immunohistochemical features of the tumor cells observed in autopsy showed the features of M4 or M5; the tumor cells displayed myelomonocytic morphology and were positive for monocyte markers CD33, CD163, and CD68 and myelocytic marker MPO. It is well known that tumor cells show features of M4 or M5 when acute transformation of CMML occurs [9]. However, we could not definitely conclude the existence of CMML in the present case, since this case did not satisfy some of the diagnostic criteria for CMML. The criteria for CMML include persistent peripheral blood monocytosis and exclusion of some genetic abnormalities such as BCR-ABL1 fusion, PCM1-JAK2 fusion, and rearrangement of PDGFRA, PDGFRB, and FGFR1 [4]. No molecular examination was conducted in this case, and since the patient had not taken health check for a while, we do not know the persistence of his monocytosis.

CMML was previously considered a subtype of myelodysplastic syndrome in the initial FAB classification [10], but in 1994, the FAB group divided it into two types, “proliferative” (CMML-myeloproliferative type) and “dysplastic” (CMML-myelodysplastic type), based on peripheral blood WBC count [11]. In 2001, the World Health Organization (WHO) subsequently classified CMML in the group of myelodysplastic syndrome/myeloproliferative neoplasms (MDS/MPNs) and is now no longer divided into proliferative and dysplastic subtypes [12]. CMML progresses to AML in up to one-third of cases, and the CMML-derived AML is now classified as AML with myelodysplasia-related changes (MRC) together with other secondary AML cases associated with MDS/MPNs or MDS [13].

The present case showed obstructive jaundice due to the pancreas mass as its initial symptom. Several swollen lymph nodes were seen near the mass at pancreatic head in this case; hence, it is possible that the pancreatic parenchyma may be infiltrated from the tumor in the peripancreatic lymph nodes. MS of the pancreas is rare. According to Norsworthy et al., only five cases of MS of the pancreatic head without a previous hematologic disorder have been reported in the English literature [3, 14–17]. All five cases were diagnosed with MS while alive, and they underwent chemotherapy with or without radiation therapy or bone marrow transplantation. Among the five cases, morphologic response of the pancreatic mass was observed in three cases upon completion of chemotherapy, suggesting the importance of early and accurate diagnosis.

No chemotherapy was administered in the present case because the biopsy specimen did not contain tumor cells and the patient's condition was rapidly deteriorating due to the PTTM-like condition. Autopsy specimens from the ampulla of Vater showed massive tumor cell infiltration to the ampullary site with occasional edematous areas without tumor cells, suggesting that the biopsy specimens might have been obtained from nontumorous sites.

PTTM is a state in which tumor cells embolize to the pulmonary vasculature resulting in the activation of the coagulation cascade, pulmonary hypertension, and DIC. PTTM is most commonly associated with gastric adenocarcinoma but has been described with numerous other carcinomas. To our knowledge, PTTM associated with a hematologic malignancy has not been reported except for one case of myelodysplastic syndrome [18]. Although the pathologic findings of the patient's lungs and the fact that he developed DIC are compatible with PTTM, our diagnosis in the present case was a PTTM-like condition because whether the patient had pulmonary hypertension or not is unknown.

Regarding the subtyping of leukemia, analysis of cell surface antigens by flow cytometry and molecular data of bone marrow specimens are usually utilized. However, in the present case, since we could not perform a biopsy of the bone marrow due to the patient's severe respiratory condition, we attempted subtyping by microscopic/immunohistochemical examination of autopsy material and the peripheral blood smear. The present case showed some confusing results in immunohistochemistry; the tumor cells were completely negative for CD34, an early hematopoietic antigen, which is known to be positive for the majority, but not all of AML [13]. For example, M5 is known to often lack CD34 expression [19].

In conclusion, we present a case of MS of the pancreatic head accompanied by a PTTM-like condition. Further attention may be warranted for our findings that [1] the cause of obstructive jaundice/pancreas mass may be hematologic malignancy and (2) immunohistochemistry for pathological specimens obtained during autopsy and smear/flow cytometry data of peripheral blood may help subtype leukemia.

## Data Availability

This case report data used to support the findings of this study are included within the article.

## Abbreviations

MS: Myeloid sarcoma
AML: Acute myeloid leukemia
CMML: Chronic myelomonocytic leukemia
MDS: Myelodysplastic syndrome
DIC: Disseminated intravascular coagulation
PTTM: Pulmonary tumor thrombotic microangiopathy
WBC: White blood cell
PLT: Platelet
CRP: C-reactive protein
CT: Computed tomography
MRCP: Magnetic resonance cholangiopancreatography
FAB: French-American-British
MDS/MPN: Myelodysplastic syndrome/myeloproliferative neoplasm
MRC: Myelodysplasia-related changes.
